# Using Pareto points for model identification in predictive toxicology

**DOI:** 10.1186/1758-2946-5-16

**Published:** 2013-03-22

**Authors:** Anna Palczewska, Daniel Neagu, Mick Ridley

**Affiliations:** 1 Department of Computing, University of Bradford, Richmond Road, Bradford, BD7 1DP, UK

**Keywords:** Predictive toxicology, Model identification, Pareto optimality, Model combination

## Abstract

Predictive toxicology is concerned with the development of models that are able to predict the toxicity of chemicals. A reliable prediction of toxic effects of chemicals in living systems is highly desirable in cosmetics, drug design or food protection to speed up the process of chemical compound discovery while reducing the need for lab tests. There is an extensive literature associated with the best practice of model generation and data integration but management and automated identification of relevant models from available collections of models is still an open problem. Currently, the decision on which model should be used for a new chemical compound is left to users. This paper intends to initiate the discussion on automated model identification. We present an algorithm, based on Pareto optimality, which mines model collections and identifies a model that offers a reliable prediction for a new chemical compound. The performance of this new approach is verified for two endpoints: IGC50 and LogP. The results show a great potential for automated model identification methods in predictive toxicology.

## Background

Predictive toxicology is concerned with the development of models that are able to predict the toxicity of chemicals [1]. These models are continuously built and validated on large collections of toxicological experimental studies to discover new biologically active compounds that are more effective, selective, less toxic, or satisfy various toxicological criteria [2,3]. A reliable prediction of toxic effects of chemicals in living systems is highly desirable in domains such as: cosmetics, drug design or food safety. This knowledge allows an earlier rejection of those chemicals that may fail the testing phase and reduces the cost of manufacturing chemical compounds in the development stage. Additionally, the European Commission’s Legislation of Registration, Evaluation and Authorization of Chemicals (REACH) [4] allows the registration of chemicals that were developed using *in silico* modelling, which facilitates a reduction in the number of animal tests. These two factors have contributed to increased interests from research and business communities in development of toxicological modelling systems that are focused on data integration, model development and predictions (e.g OpenTox [5], InkSpot [6] or OCHEM [7]).

Quantitative Structure-Activity Relationship (QSAR) or Structure-Activity Relationship (SAR) models (both regression and classification) are the most common and widely used methods to relate chemical structure/properties with their biological, chemical or environmental activities [8]. According to the Organisation for Economic Co-operation and Development (OECD) Principles for QSAR Model Validation [9], a model should be statistically significant and robust, have its application boundaries defined and be validated by an external dataset [10,11]. A model applicability domain [12,13] determines the boundary of the chemical sub-space where the model makes reliable prediction for a given activity. Applying models for chemicals from outside of their applicability domains increases the likelihood of inaccurate prediction.

There is an extensive literature associated with the best practice of model generation and data integration [14-19] but management and identification of relevant models from available collections of models is still an open problem. In recent years a large number of highly predictive models, having various applicability domains, has become publicly available. Some of them, tested on a wide chemical space, have become officially approved tools, e.g. KOWWIN (estimates the log octanol-water partition coefficient) or BCFBAF (estimates fish bioconcentration factor) built into Estimation Program Interface (EPI) Suite [20]. There is also a large number of quality models that are applicable only for a narrow chemical space. Some of them are annotated according to the OECD principles and publicly available in databases like JRC QSAR Models Database [21]. This database includes reports of model generation, validation and prediction according to the OECD standards. QSAR Model Reporting Format (QRMF) and QSAR Prediction Reporting Format (QPRF) have been developed at the Computational Toxicology and Modelling lab of the JRC’s Institute to standardise annotation of model meta-information. Currently, there is a lot of effort to build the ontologies for QSAR experiments and to provide an interoperable and reproducible framework for QSAR analyses [22].

Models that are stored in model databases can be reused to predict toxicity of new chemical compounds. Unfortunately, this involves a manual process of model identification. A potential user is required to make a comparison of model applicability domains and their predictivity for a given activity in order to decide if the model can make reliable predictions for a given chemical compound. Model comparison is a difficult task since models are generated using various subsets or various chemical compound descriptors. Consequently, models can be trained and validated on different datasets. For regression models, the model performance can be described by the predictive squared correlation coefficient *q*^2^. Since the sizes and contents of modelling and validation datasets may differ for various models, the value of *q*^2^ is not sufficient for model comparison [10]. Several model performance matrices were analysed in the context of model validation and model selection [14]. They are applied in automated model development where models are validated by the same dataset. In the case where two models come from different sources, model comparison becomes challenging. This requires predictive models to be validated across the entire chemical space, which is very difficult as the list of available chemicals and assays may be limited.

Clearly, there is a need for automated techniques for mining model repositories. This includes methods for model quality control, data and model integration, model comparison and model identification. Our research aims to address this gap. In this paper, we draw attention to the importance of existing models’ usage in predictive toxicology. We also introduce methods for effective model identification for a new unseen chemical compound. The term “model identification“ covers the whole range of problems related to model selection from a collection of models (for a given endpoint) developed on various datasets. In the extreme case, datasets (and specified applicability domains) for two models can be disjoint. Model identification is a much harder problem than the well known model selection problem [23], i.e choosing a model from a set of candidate models with the same applicability domains. Therefore, various methods applied in traditional model selection [24-27] cannot be directly applied to model identification. In contrast to model selection, model identification cannot take into account model variables or parameters since some model variables cannot be easily accessed for new chemical compounds.

The interesting questions here are whether efficient model identification is possible based on molecular structures and models performances, and how good the identified model can be for a new chemical compound. In [28], authors defined the framework for automated model selection and described a simple algorithm for model selection. The method selects the most predictive model from the collection of models for a nearest neighbour to the query chemical compound. Often, the nearest neighbourhood can contain more than one element and model performances can differ slightly. In this case, it is difficult to say which model would be the most reliable for a given chemical compound.

To answer the above question, in this paper we present a new method for model identification for regression models. This method uses Pareto points [29] to define the nearest Pareto neighbourhood according to two criteria: structural similarity of chemicals and models performances. In the next section a framework for model identification, Pareto points and their properties are introduced. Having the Pareto nearest neighbourhood defined, we present two methods for model identification. The first method averages model performances for all Pareto neighbours and identifies the one with the smallest error. The second method identifies a model for which the Pareto point is the closest (based on Euclidean distance) to a centroid of all points in the Pareto neighbourhood. We also demonstrate that model identification improves the quality of the test set, or unseen chemical compound prediction. Experimental work using IGC50 for Tetrahymena pyriformis and internal Syngenta LogP datasets show that our approach provides good results and it is worth being considered for further research.

## Methods

### Framework for model identification in predictive toxicology

There are several chemical compound representations and thousands of available chemical descriptors [8] used for predictive model development. In this paper, a *chemical spaceX* is a set of chemicals represented by pairs *x* = (*x*^*d*^,*x*^*f*^), where $x^{d}\in R^{K_{1}}$ represents a vector of descriptor values, $x^{f}\in {\left\{0,1\right\}}^{K_{2}}$ is a fingerprint, and *K*_1_ + *K*_2_ is the dimension of the chemical space. Descriptors represent various topological, geometrical, physical and chemical properties of a chemical compound. A fingerprint is a binary vector whose coordinates define the presence or absence of predefined structural fragments within a molecule [30]. A fingerprint is also a one dimensional representation of a chemical compound and it is widely used for chemical similarity search in large databases [31]. It is also worth noting that a fingerprint is not a unique chemical compound representation because it encodes only a fragment of a molecule. There can be two different molecules having the same fingerprint representation.

A *predictive model M *is a mapping X → Y, where Y⊂R is the output space. The output space *Y* might, for example, represent a particular biological, physical or chemical activity of a chemical compound.

The input data is represented by the pairs: (*x*_*i*_,*y*_*i*_) ∈ *X* × *Y* for *i* = 1,…,*n*, where *x*_*i*_ is an element of the chemical space and *y*_*i*_ is the measured activity of that element. There is also a set of *m*  predictive models $ℳ=\{M_{1},\ldots ,M_{m}\}$ associated with the activity *Y*. These models were generated using various statistical or data mining techniques and they have different applicability domains and performances. To identify the most predictive model from the collection of models ℳ for a new chemical compound *x*, we define a *partitioning model* that splits the chemical space into disjoint groups and allows an unambiguous model identification.

A *partitioning model*$\hat{M}$ is a mapping *X* → *Y* given by the following formula:

(1) $$\hat{\;M}(x)=\left\{\begin{array}{cc} M_{1}(x), & x\in D_{1},\\ M_{2}(x), & x\in D_{2},\\ \vdots & \vdots \\ M_{m}(x), & x\in D_{m}, \end{array}\right)$$

where 

• *D*_1_,…,*D*_*m*_ ⊆ *X* are disjoint,

• $\overset{m}{\underset{i=1}{⋃}}D_{i}=X$.

The main hypothesis in predictive modeling is that similar chemical compounds have similar properties [32]. Following this hypothesis we build the partitioning model that it splits the chemical space in groups in order to maximize the similarity of their chemical compounds and to minimize the error of a model associated with this group. It is easy to notice that this is a bi-criteria problem and the solutions have to represent a trade-off between optimality of these criteria (the so-called Pareto points). Pareto optimality is a multi-criteria optimisation technique widely applied in decision making problems [29]. In QSAR modelling multi-objective (criteria) was used for feature selection [33] in order to maximize predictive capacity and to reduce the number of selected descriptors. In this paper we present how Pareto optimality can be applied in QSAR model identification. In the following sections we recall the basic definition of the Pareto set and we propose an algorithm that finds Pareto points in 2D vector space.

### Pareto points and their properties

Let consider a vector $v=\;[f_{1},f_{2},\ldots ,f_{K}]$ in the *K*– dimensional space. Let *π*_*j*_(*v*) = *f*_*j*_ denote a *j*-th coordinate of vector *v* and *V* be a finite set of vectors in $R^{K}$.

#### Definition 1 (Domination)

A vector $v\in R^{K}$ is dominated by a vector $w\in R^{K}$, which is denoted by *v* ≼ *w*, if

(2) $$\pi_{j}(v)\leq \pi_{j}(w),\;\forall j=1,\ldots ,\mathrm{K.}$$

We say that *v* is strictly dominated by *w* (*v* ≺ *w*), if *v* ≼ *w* and *v* ≠ *w*, i.e.

(3) $$\;\forall j=1,\ldots ,K\;\pi_{j}(v)\leq \pi_{j}(w),\;\exists_{j=1,\ldots ,K}\;\pi_{j}(v)<\pi_{j}(w).$$

#### Definition 2 (Comparison)

Vectors $v,w\in R^{K}$ are incomparable, which we denote by *v*∼*w*, if neither *v* ≼ *w* nor *w* ≼ *v*.

Note that *v* ∼ *w* if and only if there exist i,j∈{1,…,K}, *i* ≠ *j*, such that

(4) $$\pi_{i}(v)<\pi_{i}(w)\;\text{and}\;\pi_{j}(v)>\pi_{j}(w).$$

#### Definition 3 (Pareto set)

A set *Γ* ⊂ *V* of minimal vectors with respect to ≼ is called a Pareto set for *V*.

Note that *Γ* consists of incomparable vectors. We can define *Γ* equivalently by the formula

(5) $$\Gamma =\{v\in V:\forall_{w\in V}\;v≼w\vee v∼w\}.$$

The above definitions and basic properties of the Pareto set can be found in [34]. Now, we introduce below some properties of Pareto sets and Pareto order that are used in the following sections. First, we introduce the convenient notation. Let

(6) $$f_{j}^{\text{min}}:=\min\{\pi_{j}(v):v\in V\},\;j=1,\ldots ,K,$$

and

(7) $$V_{j}:=\{v\in V:\pi_{j}(v)=f_{j}^{\text{min}}\},\;j=1,\ldots ,\mathrm{K.}$$

The set *V*_*j*_ consists of all vectors in *V* with minimal value on the *j*-th coordinate.

#### Lemma 1

Let *Γ*_*j*_ be the set of all minimal vectors in *V*_*j*_. Then *Γ*_*j*_ ⊂ *Γ*, where *Γ* is the Pareto set for *V*.

Let $I\Gamma =\underset{j=1,\ldots ,K}{⋃}\Gamma_{j}$ and

(8) $$f_{j}^{\text{max}}:=\text{max}\{\pi_{j}(v):v\in I\Gamma \},\;j=1,\ldots ,\mathrm{K.}$$

In particular, I*Γ* is a subset of *Γ* and it is called an initial Pareto set. Now we establish the dependence of the conditions for incomparability with vectors in this initial Pareto set.

#### Lemma 2

If a vector *v* ∈ *V* is incomparable with all vectors in I*Γ*, then there exist at least two indices j∈{1,…,K} such that

(9) $$\pi_{j}(v)\in (f_{j}^{\text{min}},f_{j}^{\text{max}}).$$

The proof of this Lemma 1 and Lemma 2 as well as all other results in the paper are provided in Appendix 1.

### Pareto order in two dimensions

This subsection is devoted to the study of the two-dimensional case, i.e. *K* = 2. We shall use the notation introduced above.

#### Lemma 3

The set I*Γ* has at most two elements. 

1. If |I*Γ*| = 1, then I*Γ* is the Pareto set for *V*. 

2. If |I*Γ*| = 2, then a vector *v* ∈ *V* is incomparable with vectors in I*Γ* if and only if

(10) $$\forall_{j=1,2}\;\pi_{j}(v)\in (f_{j}^{\text{min}},f_{j}^{\text{max}}).$$

**Figure 1:**
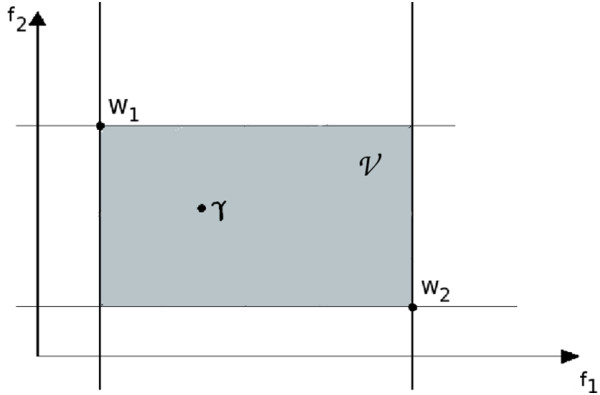
**A space **V **of incomparable vectors bounded by coordinates vectors w**_**1**_**,w**_2_ ***∈*** **I*Γ*.**

**Figure 2:**
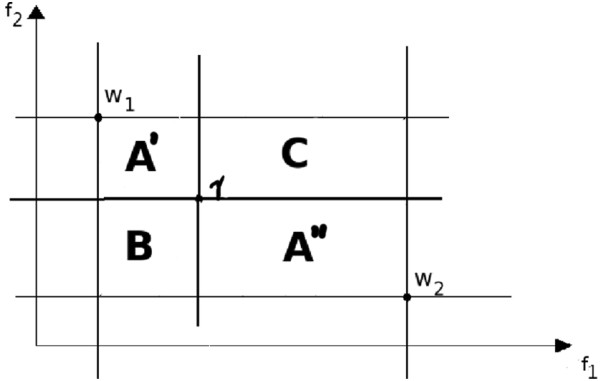
**A partition of space **V **when a new vector *γ*** **is introduced.**

As shown in Figure 1 and Figure 2, when I*Γ* consists of two elements *w*_1_ and *w*_2_, a set of vectors incomparable with I*Γ* is given by the rectangle V. Let *Γ* be a vector incomparable with I*Γ*, i.e. γ∈V. The introduction of *v*_0_ divides the rectangle V into three areas: 

• *A*^′^ and *A*^′′^ is a set of vectors incomparable with I*Γ* ∪ {*γ*},

• *B* is a set of vectors smaller then *Γ*,

• *C* is a set of vectors bigger then *Γ*.

The above properties of I*Γ* and vectors incomparable with I*Γ* allow us to limit the search spaceVto find Pareto solutions.

### Finding a Pareto set in 2D vector space

In this section, we present an algorithm for finding a Pareto set in two-dimensional space (see Algorithm 1). FIND-PARETO-SET(*V*) is a recursive algorithm that finds all Pareto points in the rectangleV defined by two points in the initial Pareto set I*Γ* (see Lemma 1); this rectangle contains all points from *V*. The algorithm starts from finding a point *Γ* that does not dominate any other points in *V* (line 4). This point splits the areaV into four rectangles (see Figure 2). According to Lemma 2 and Lemma 3, **B** ∩ *V* = *∅*, **C** does not contain Pareto points, whereas points in rectangles **A**^′^ and **A**^′′^ are incomparable with *Γ*. The above procedure is recursively repeated for **V** ∩ **A** ^′^ and **V** ∩ **A**^′′^.

#### Algorithm 1 **FIND-PARETO-SET**(***V***)

The algorithm sketched above calls FIND-PARETO-POINT($\bar{V}$) (see Algorithm 2) to find a Pareto point in the set$\bar{V}$. This procedure works in the pessimistic time *O*(*n*^2^), where *n* is a number of elements in$\bar{V}$ (when all solutions are comparable, i.e., to form a chain it may take *n*iterations to find a Pareto point). However, the expected running time is much shorter thanks to the random selection of points.

#### Algorithm 2 **FIND-PARETO-POINT**($\bar{V}$)

### Model identification in predictive toxicology

Following the similarity hypothesis researchers build models for groups of chemicals that have a common molecular fragment or common properties. These models are more reliable and give better predictions for chemicals that lie in the model applicability domains. Further, high quality models developed for a small subset of chemical space can be combined in a global model that covers larger chemical space using various ensemble techniques. In this section we present how to identify a reliable model from a collection of already existing models for new before unseen chemicals.

**Figure 3:**
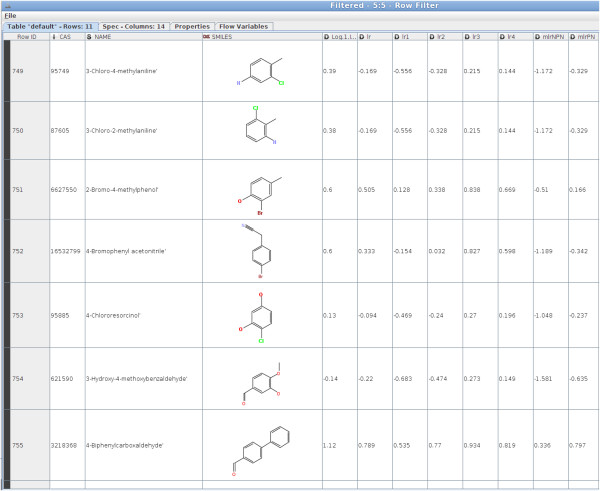
**Collection of models for the IGC50 prediction for Tetrahymena pyriformis.** The first three columns include chemical compound representation. The fourth column represents the measured value of IGC50. The presentation of model predictions starts from the fifth column.

The chemical space *X* is a set of chemical compounds represented by the combination of all possible existing chemical descriptors, and for a given endpoint there is a collection of existing modelsℳ. For each chemical compound *x* ∈ *X*, model predictions$Y^{\prime }=\{y_{1}^{\prime },\ldots ,y_{m}^{\prime }\}$ for models fromℳ are known (see Figure 3). To identify a model for a given query chemical compound *q* we convert the set of chemicals from X and their model performances into a set of pairs (*d*_*i*_,*e*_*im*_), where *d*_*i*_ represents the distance between *q* and the *i*–th chemical compound from the chemical space. The error$e_{\text{im}}=|y(x_{i})-y_{m}^{\prime }(x_{i})|$ defines the model performance for the *m*–th model fromℳ and for the *i*–th chemical compound. In a set of such pairs, one can find models that have a low predictive power for the most similar chemical compounds whereas the other gives better predictions. This illustrates the situation often encountered in multicriterial optimization problems: there is no solution that outperforms the others with respect to all criteria. Hence, instead of having one solution we have a set of solutions that cannot be compared to each other. The above task is a Pareto problem: one has to balance similarity to existing chemical compounds and correctness of predictions offered by available models.

The model identification procedure (see Algorithm 3) can be described as follows: for a query chemical compound *q* and a given chemical space – 1) create the set *V* of pairs (*d*_*i*_,*e*_*im*_), 2) find the Pareto set for *V*, 3) select the most suitable model for *q*. To create a set *V* we start from the array *T* (see Figure 3) that contains a structural representation of the chemical compound, its measured activity (for a given endpoint) and predictive performance of each model from ℳ.

#### Algorithm 3 **MODEL-IDENTIFY**(***Tq***)

After executing MODEL-IDENTIFY(*T*,*q*), in line 1, the array *T* is converted into a list of vectors *V* using procedure INIT(*T*,*q*) (see Algorithm 4). Every vector *v*_*i*_ ∈ *V* is defined as a pair of the distance between *q* and the *i*-th chemical compound from *T*, and the error of the *j*-th model fromℳfor the compound *i*. The distance *d*_*qi*_ = 1 - *ST*_*qi*_ is calculated using Tanimoto coefficient *ST*, which is the most frequently used similarity measure in chemoinformatics [35]. This coefficient works with fingerprints (binary representation of molecules) and is defined as a ratio between the number of bits set on the same position in two fingerprints and the sum of bits set on different positions. The model error *e*_*ij*_ is defined as a distance between the true activity for compound *i* and the value computed by model *j*. We treat *V* as a set of all possible solutions for model identification for a given query molecule *q* and known chemical sub-space.

#### Algorithm 4 **INIT**(***T***,***q***)

In line 2 of MODEL-IDENTIFY(*T*,*q*), we call FIND-PARETO-SET(*V*) to find the set of all Pareto points *Γ* in *V*. Then, we analyse points in *Γ* in order to choose the most predictive model for *q*. In the case when |*Γ*| = 1, there is only one candidate, so the choice is trivial. This case is comparable to the algorithm proposed in [28] which selects the most predictive model for the most similar chemical compound of *q*. In the case when *Γ* consists of many Pareto points, the model identification becomes a difficult task: the Tanimoto similarity coefficient (as well as other fingerprint similarity measures) between chemical compounds may not be correlated enough with their activity partially contradicting the similarity hypothesis [32] (see the end of this section for a detailed example). To identify a model using Pareto points, first we define *n*-Pareto Neighbourhood as follows:

#### Definition 4

*n*-Pareto Neighbourhood is a set with at most *n* - Pareto points from *Γ* which are at distance less than *τ* from the element *q* where *τ* > 0 and *n* > 0.

The threshold *τ* is selected by experiment and depends on the chemical similarity within a given chemical space. Having defined the Pareto neighbourhood for a given chemical compound *q*, we provide two methods for model identification. The first one is called *n*-Average Pareto (see Algorithm 5). The threshold *τ* provides means for removing those chemical compounds which are dissimilar to the query compound *q*  but their activity is very well predicted by some model. Next, the model average model errors for the chemicals represented by Pareto points and then the model with the smallest average error is selected. We call this method *n*-Average Pareto Model Identification (*n*-APMI). The usage of Pareto neighbourhood in comparison with the standard nearest neighbourhood is that this method is more sensitive on model performances and allows for the rejections of the similar chemical compounds on which models perform badly.

#### Algorithm 5 **Average Pareto**

The second method is called *n*-Centroid Pareto (see Algorithm 6). For all Pareto points from the *n*-Pareto Neighbourhood the centroid Pareto point *c* is calculated according to formula:

(11) $$c=(d_{c},e_{c})=\left(\right)\frac{\underset{p\in n-\text{PN}}{\sum }d_{p}}{\left|n-\text{PN}\right|},\frac{\underset{p\in n-\text{PN}}{\sum }e_{p}}{\left|n-\text{PN}\right|})),$$

where *d*_*c*_ is the average of distances and *e*_*c*_ is the average of model errors for all Pareto points from the neighbourhood (*n* - *PN*). In the next step the Euclidean distance between Pareto points and the centroid is computed. The model that is associated with the Pareto point for which the Euclidean distance to the centroid is minimal, is selected. We call this method *n*-Centroid Pareto Model Identification (*n*-CPMI). According to the definition, both *n*-APMI and *n*-CPMI are partitioning models that splits chemical space into disjoin groups and allow unambiguous model identification.

#### Algorithm 6 **Centroid Pareto**

We mentioned above that similar chemical compounds might have very different measurements of activity. To demonstrate this, we analysed the TETRATOX [36] dataset which contains growth inhibition concentration (IGC50) for Tetrahymena pyriformis. Chemical compounds were compared in pairs. Their Tanimoto similarity coefficient and differences in measured activity were collected. Summarised results are displayed in Table 1. Column headers hold differences in the measured activity between two chemicals, while row headers describe molecule similarity threshold. The single cell of this array represents a number of pairs of chemical compounds for which the distance is smaller than the row identifier and the difference in the activity is smaller than the column identifier.

**Table 1:** Analysis of chemical compound similarities in order to highlight the difference of the chemical activity for the TETRATOX dataset

***f***_***sim***_**/*diff***_***activ***_	**0**	**0.1**	**0.2**	**0.3**	**0.4**	**0.5**	**0.6**	**0.7**
0	1	2	2	2	2	2	2	2
0.1	3	13	27	44	51	62	70	79
0.2	6	112	220	335	431	512	585	655
0.3	16	318	617	933	1213	1474	1719	1928
0.4	32	720	1402	2081	2701	3297	3840	4328
0.5	66	1380	2726	4042	5227	6437	7536	8547
***f***_***sim***_**/*diff***_***activ***_	**0.8**	**0.9**	**1**	**1.1**	**1.2**	**1.3**	**1.4**	**1.5**
0	2	2	2	2	2	2	2	2
0.1	84	90	93	96	99	103	104	104
0.2	700	753	782	801	827	842	849	858
0.3	2106	2278	2412	2507	2621	2715	2784	2821
0.4	4763	5160	5526	5837	6119	6360	6575	6724
0.5	9481	10362	11167	11840	12488	13082	13589	14004

The TETRATOX dataset contains over one thousand chemical compounds and the biggest difference between measured values of IGC50 is equal to 5.3. Notice that the number of pairs of chemicals that are similar, based on both the fingerprint similarity and the activity, is very small. There is only one pair of chemical compounds that have the same activity and maximal similarity (1-row, 1 column). On the other hand, there are many chemicals which are similar fingerprint-wise but have different activities. This makes the model identification challenging.

In the next section we present results of the experiments that were carried out in order to demonstrate how model identification works.

## Experimental results

Two experiments were proposed in order to demonstrate the advantages of model identification for predictive toxicology. Each experiment has two phases. In the first phase we treated model identification as a classification problem to study the performances of proposed methods in comparison with the other classification algorithms. We defined an “oracle model” that associates each chemical compound from a given chemical space with the most predictive model from the collection of existing models and we used this model to validate our methods. In the second phase, for each chemical compound we applied an identified model to predict the growth inhibition concentration (IGC50) in the first experiment and Partition coefficient (LogP) in the second. Finally, we compared these results with the original model performances applied to the whole chemical space.

### IGC50 Prediction for Tetrahymena Pyriformis

A dataset (Tetrahymena Pyriformis Toxicity - TPT) of 1129 chemicals was obtained from the INCHEMICOTOX webpage [37]. This dataset is compiled of toxicity data for the unicellular ciliated protozoa Tetrahymena pyriformis (see [38]) and was published in [39]. The measure of toxicity is 50% growth inhibition concentration (IGC50). Two QSAR regression models were obtained from INCHEMICOTOX. These models are also reported in the JRC QSAR Models Database. The first, non polar narcosis (NPN) QSAR [40], was originally trained on 87 chemicals identified as non polar narcotics with *q*^2^ = 0.95. The linear regression model was defined as follows:

log(1/IGC50)=0.83logP-2.07,

where log *P* is the octanol-water partition coefficient. The second, polar narcosis (PN) QSAR model [41] for Tetrahymena pyriformis, was trained on 138 polar narcotics chemicals with *q*^2^ = 0.75 and defined as follows:

log(1/IGC50)=0.62logP-1.00.

Training datasets for both models were obtained from JRC QSAR Models Database. These datasets were compared with the Tetrahymena pyriformis dataset and 204 (136 from the PN model and 68 from the NPN models) training chemicals were present in the TPT dataset. We did not perform any data curation for this dataset. The above described models were implemented for the log*P* value calculated using the cdk library [42] and used to predict toxicity for the TPT datasets.

First, we considered the model identification problem as a classification problem to predict which model will be the most reliable for a given chemical compound. Having a dataset of the predicted IGC50 for both models and the measured value, we used *a priori* information (“oracle model“) about the best selected model for each chemical compound and we applied various classification methods. To simulate the model identification for before unseen chemical compounds the leave-one-out (LOO) method was used. This methods takes out one chemical compound from the dataset and uses others chemicals to predict which model would be the most reliable for it. This procedure were repeated for all chemicals in the dataset.

Table 2 includes results from the comparison of *n*-CPMI and *n*-APMI proposed in this paper with the DMS (Double Min Score algorithm) [28] and with the standard classification algorithms such as: NaiveBayes, BayesNet decision trees (PART and J48), nearest neighbour (IBK) or support vector machine (SMO) implemented in WEKA [43]. These classifiers were initialised by the default parameter settings. The dataset, used to generate these classification models, consisted of chemicals represented by binary descriptors (1024 - bit fingerprints calculated using cdk library) and the model errors. We compared all classifiers according to a number of the correctly classified chemicals and the classifiers accuracies. The 3-APMI methods gives the highest number of correctly classified elements and relatively low numbers for false positive and false negative - especially comparing this method to the IBK(3). The 3-APMI uses the 3-Pareto neighbourhood where as IBK(3) uses the 3-nearest neighbourhood for classification. This shows that the model identification using Pareto points is as good as or can be better than the other well know classification algorithms.

**Table 2:** Comparison of classification algorithms according to a number of correctly classified elements, false positive, false negative and the classifiers accuracies

**Method**	**Correct class**	**Falsepositive**	**False negative**	**Accuracy**
SMO	899	122 (10.8%)	106 (9.4%)	0.80
Part	904	123 (10.9%)	101 (8.9%)	0.80
NaiveBayes	845	191 (19%)	90 (7.9%)	0.75
J48	905	123 (10.9%)	100 (8.9%)	0.80
IBK(1)	905	121 (10.7%)	102 (9%)	0.80
IBK(3)	901	133 (11.7%)	94 (8.3%)	0.79
IBK(5)	889	149 (13.2%)	93(8.2%)	0.78
BayesNet	756	264 (23%)	108 (9.5%)	0.67
DMS	901	115 (10.1%)	112 (9.9%)	0.79
3-CPMI	902	136 (12%)	90 (7.9%)	0.79
5-CPMI	897	137 (12%)	94 (8.3%)	0.79
10-CPMI	863	168 (14.8%)	97 (8.5%)	0.76
**3-APMI**	**918**	**99 (8.7%)**	**111(9.8%)**	**0.81**
5-APMI	891	115 (10%)	122 (10.8%)	0.78

The polar narcosis model label was defined as the positive class.

**Figure 4:**
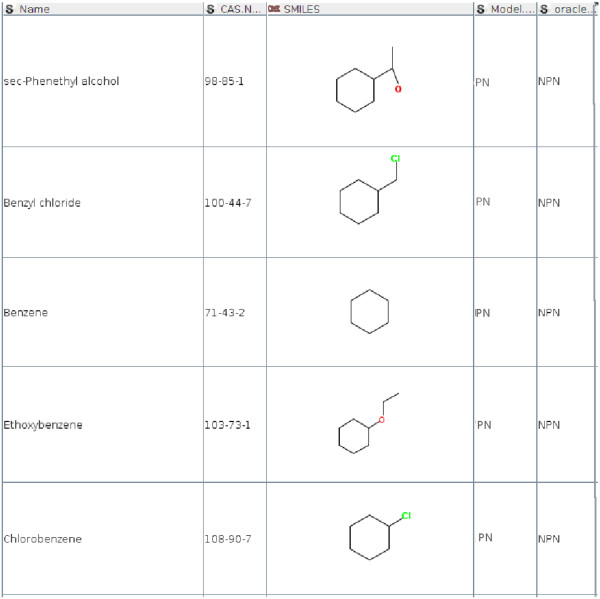
Chemical compounds wrongly associated with the PN model by 3-APMI.

**Figure 5:**
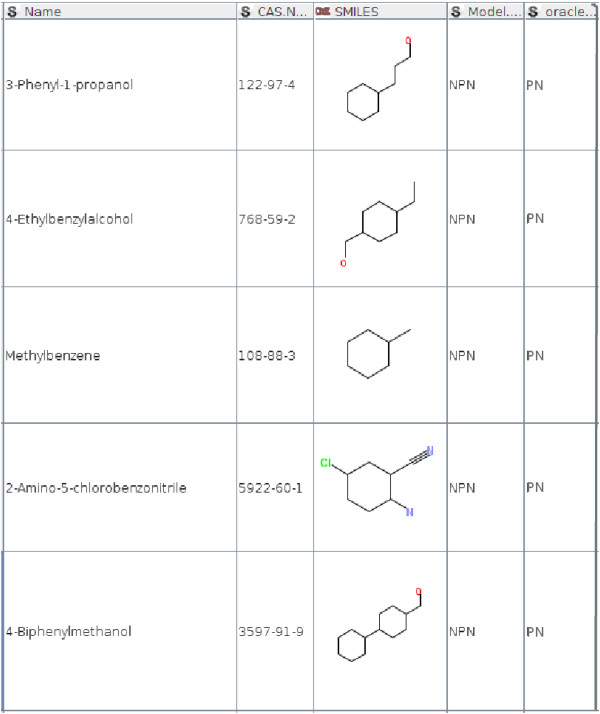
Chemical compounds wrongly associated with the NPN model by 3-APMI.

The decision on model identification relies on the distance to the Pareto points. Figures 4 and 5 show misclassification examples for the 3-APMI method. On Figure 5 for *3-Phenyl-1-propanol* the NPN model was identified. Its Pareto neighbourhood included three chemicals: *4-Chloro-3-methylphenol Methylbenzene* and *4-Dimethylbenzene* with the distances and models errors shown in Table 3. The 3-APMI model averages model errors for all Pareto points in this neighbourhood and selects the one with the smallest average, in this case the NPN model. One can notice that the best model for this Pareto neighbourhood is the NPN model for *4-Dimethylbenzene* whereas this chemical compound is not the most similar to the query chemical compound.

**Table 3:** **Model performances and distance comparison of the 3-Pareto neighbourhood of the *3-Phenyl-1-propanol***

**Name**	**Distance**	**PN**	**NPN**
Methylbenzene	0.33	0.37	0.28
4-Dimethylbenzene	0.36	0.54	0.08
4-Chloro-3-methylphenol	0.30	0.61	1.14

To demonstrate a correct classification example, we selected *Benzylamine* that was associated correctly with the PN model. Its Pareto neighbourhood included two chemicals: *2-Chloroaniline* and *(+/-)-1,2-Diphenyl-2-propanol* with distances and model performances shown in Table 4 (notice that according to Definition 4, the three Pareto neighbourhood consists of at most three Pareto points). These distances to the query chemical compound are small and for both chemicals the PN model gives the most reliable prediction. The 3-APMI identifies the PN model that has the minimal average error for all Pareto neighbours.

**Table 4:** **Model performances and distance comparison of the 3-Pareto neighbourhood of the*Benzylamine***

**Name**	**Distance**	**PN**	**NPN**
2-Chloroaniline	0.08	0.30	0.38
(+/-)-1,2-Diphenyl-2-propanol	0.11	0.041	0.59

**Figure 6:**
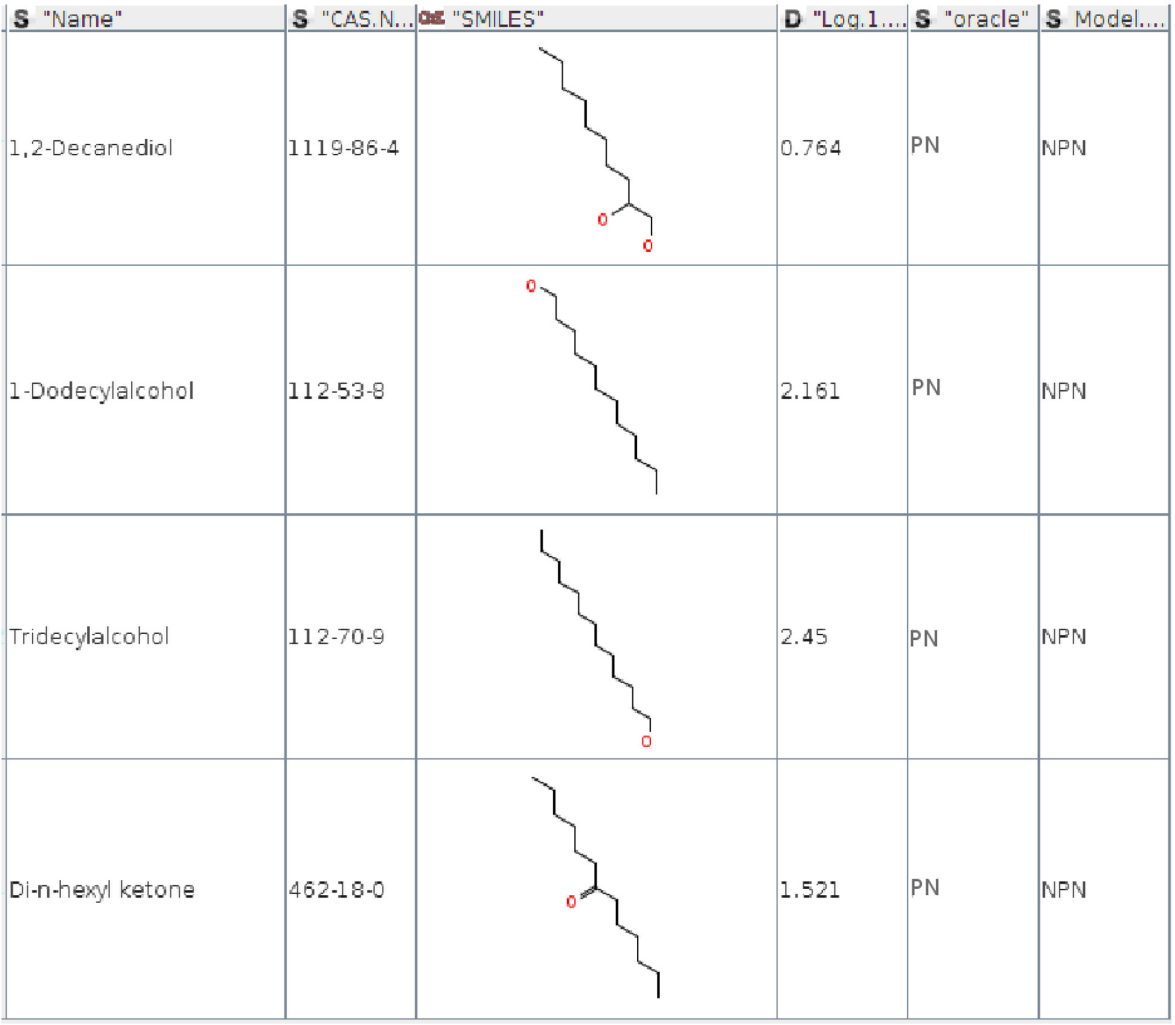
Chemical compounds that were originally used to train the NPN model but associated with the PN model by the oracle.

**Figure 7:**
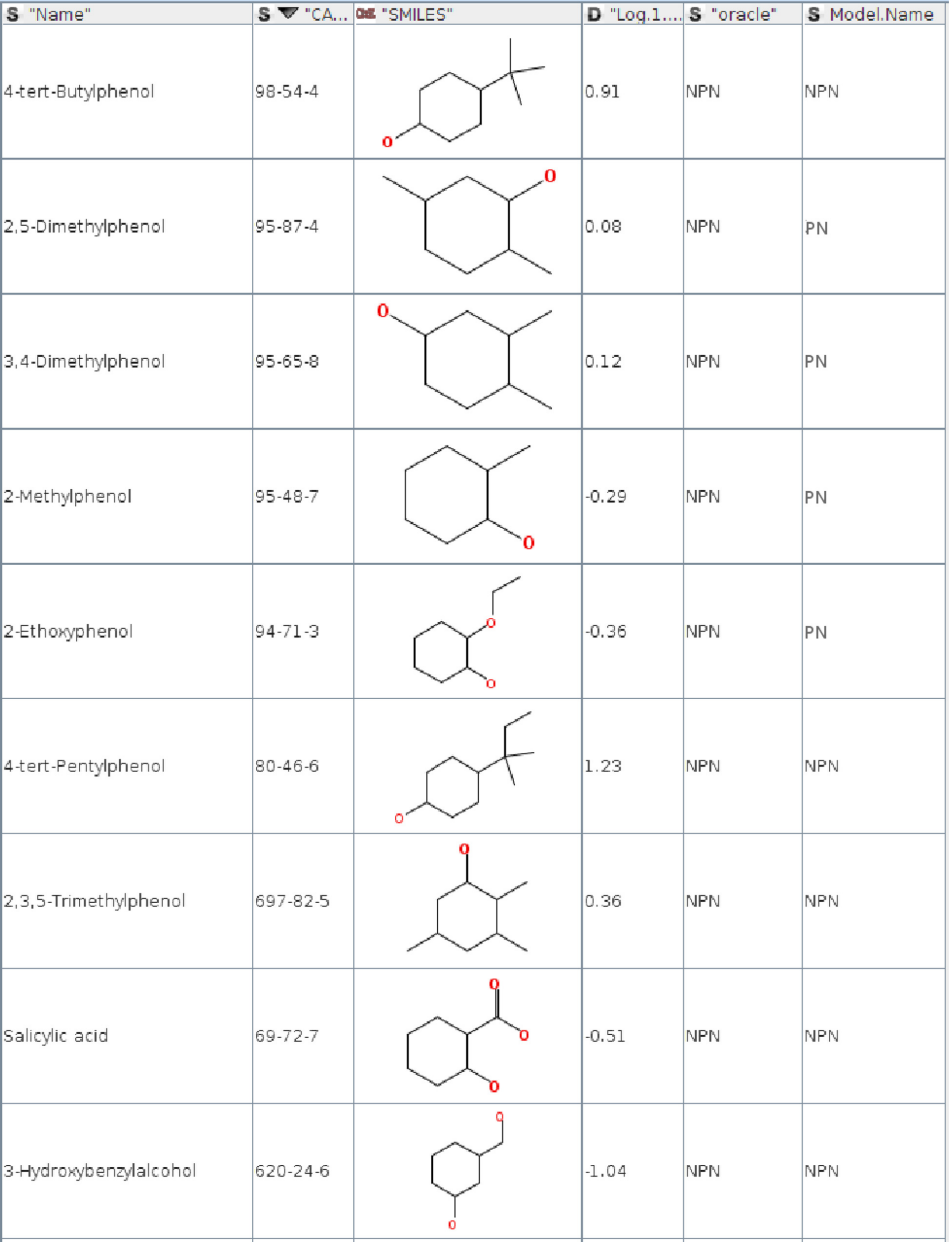
Chemical compounds that were originally used to train the PN model but associated with the NPN model by the oracle.

Additionally, from the entire TPT dataset, chemicals included in the original training datasets for both models were selected. We identified 4 out of 68 chemicals that were used to train the NPN model but the oracle model associated them with the PN model (see Figure 6). The same analysis were repeated for the training dataset of the PN model and we identified 9 out of 136 chemicals that were associated with the NPN model by the oracle model (see Figure 7).

To predict IGC50 for the TPT dataset we used the identified model for each chemical compound in this dataset. The results obtained for the entire dataset are shown in Table 5. The statistics used are: R2 - correlation coefficient for the observed and predicted values, RSE - root-squared error, Q2 - predictive squared correlation coefficient, MAE - mean absolute error and RMSE - root mean square error. The “oracle model” has the knowledge of the best model for each chemical compound. Its predictivity is low because we used only two existing models from JRC QSAR database that were designed based on mode-of-action (polar/non polar narcosis) for chemicals from TPT.

**Table 5:** Analysis of model prediction accuracies for IGC50 for Tetrahymena pyriformis

**Method Name**	**R2**	**RSE**	**Q2**	**MAE**	**RMSE**
NPN	0.58	0.66	0.15	0.69	0.94
PN	0.58	0.66	0.58	0.50	0.66
DMS	0.68	0.56	0.62	0.43	0.62
3-CPMI	0.67	0.58	0.60	0.43	0.63
5-CPMI	0.66	0.59	0.59	0.44	0.65
10-CPMI	0.65	0.60	0.57	0.44	0.66
**3-APMI**	**0.69**	**0.56**	**0.65**	**0.41**	**0.60**
5-APMI	0.68	0.57	0.62	0.42	0.62
Oracle	0.75	0.50	0.71	0.35	0.54

The 3-APMI method provides the best prediction among “non-oracle models”. The first two rows present prediction statistics for PN and NPN models. They are lower than for all other models. Notice, however, that their R2 and RSE statistics are identical. This is due to the fact that both models are affine functions of one and the same explanatory variable. An affine function can, therefore, transform one model into another. This is what happens when regression is applied to compute R2 and RSE. Notice that other two measures of Q2 and predictive errors are different for these models.

As another example, we considered only a small subset of the whole initial TPT dataset that contains only 376 chemical compounds. This dataset includes all training chemicals used in PN and NPN models plus over 100 additional chemicals from the TPT dataset. We included chemicals for which the absolute error of the oracle model is less than 0.4 and they are in the applicability domain of both models. The value of log*P* ∈ [-0.5,6.2] and the toxicity value is in the range [-2.5,3.05]. Again we compared various classifiers that were used for model identification (see Table 6).

**Table 6:** Comparison of classification algorithms according to a number of correctly classified elements, false positive, false negative and the classifiers accuracies

**Method**	**Correct class**	**Falsepositive**	**Falsenegative**	**Accuracy**
SMO	296	47(12%)	33(8.7%)	0.787
Part	303	34(9%)	39(10.3%)	0.805
NaiveBayes	281	67(17%)	28(7.4%)	0.747
J48	296	44(11.7%)	36(9.5%)	0.787
IBK(1)	307	42(11.1%)	27(7.1%)	0.816
IBK(3)	300	42(11.1%)	34(9%)	0.797
IBK(5)	299	46(12.2%)	31(8.2%)	0.795
BayesNet	273	76(20.1%)	27(7.1%)	0.726
DMS	297	48(12.7%)	31(8.2%)	0.719
**3-CPMI**	**316**	**29 (7.7%)**	**31(8.2%)**	**0.844**
5-CPMI	305	33(8.7%)	38(10.1%)	0.811
10-CPMI	288	41(10.9%)	47(12.5 %)	0.766
3-APMI	306	33(8.7%)	37(9.8%)	0.813
5-APMI	300	41(10.9%)	35(9.3%)	0.797

The polar narcosis model label was defined as the positive class.

In this case, the best method is 3-CPMI that from the 3-Pareto neighbourhood selects model for which Pareto point is the closest to the neighbourhood centroid. This method gives better results if compared with the DMS method that selects the model with the smallest error for the nearest neighbour. Tables 7 and 8 show the list of chemicals that were wrongly classified by the 3-CPMI algorithm. Comparing the regression models for IGC50 (see Table 9), 3-CMPI method provides better prediction than DMS, PN and NPN models.

**Table 7:** Chemical structures wrongly associated with the PN model by 3-CPMI

**CAS**	**Smiles**
4097498	CC(C)(C)C1=CC(=C(C(=C1)[N+](=O)[O-])O)[N+](=O)[O-]
6920225	CCCCC(O)CO
928972	CCC=CCCO
10031875	CCC(CC)COC(=O)C
112141	C(C)(=O)OCCCCCCCC
105668	C(CCC)(=O)OCCC
624544	O(C(CC)=O)CCCCC
123660	C(CCCCC)(=O)OCC
123159	CCCC(C=O)C
2987168	CC(C)(CC=O)C
96480	O=C1CCCO1
19686738	CC(CBr)O
4620706	C(NCCO)(C)(C)C
111864	CCCCCCCCN
597977	C(N=C=S)(C)(C)CC
17112822	c1c2c(CN=C=S)cccc2ccc1
1138529	CC(C)(C)C1=CC(=CC(=C1)O)C(C)(C)C
142303	C(#CC(C)(C)O)C(C)(C)O
31333138	CCCCCC#CCCO
107879	CC(CCC)=O
2067336	OC(CCCCBr)=O
91156	N#Cc1c(C#N)cccc1
2065238	c1(ccccc1)OCC(OC)=O
613978	N(CC)(C)c1ccccc1
586787	[N+](c1ccc(cc1)Br)(=O)[O-]
91667	c1(N(CC)CC)ccccc1
38713563	O(CCCCCCCCC)C(=O)c1ccc(O)cc1
622468	C(Oc1ccccc1)(=O)N
93914	C(CC(=O)C)(=O)c1ccccc1
2216946	C(#Cc1ccccc1)C(=O)OCC

**Table 8:** Chemical structures wrongly associated with the NPN model by 3-CPMI

**CAS**	**Smiles**
29338496	CC(C(C1=CC=CC=C1)C2=CC=CC=C2)O
100447	C1=CC=C(C=C1)CCl
1823912	CC(C#N)C1=CC=CC=C1
103695	CCNC1=CC=CC=C1
112538	C(CCCCCCCCCCC)O
1119864	C(CCCCCC)CC(CO)O
628637	C(C)(=O)OCCCCC
108225	O(C(=C)C)C(=O)C
94042	C(C(OC=C)=O)(CCCC)CC
1932929	C(CC)(=O)OCC#C
1732098	O(C(CCCCCCC(OC)=O)=O)C
110623	C(CCCC)=O
36536466	O=C1CC(C)O1
6261229	CCC#CCO
4753597	O(CCCCBr)C(C)=O
20965279	N#CCCCCCCBr
1577180	OC(=O)CC=CCC
111160	C(CCCCCC(=O)O)(=O)O
535137	C(C(C)Cl)(=O)OCC
600000	CCOC(=O)C(C)(C)Br
23165448	c1ccc(CCCC)cc1N=C=S
1565759	CCC(C)(C1=CC=CC=C1)O
529191	CC1=CC=CC=C1C#N
141286	C(CCCCC(OCC)=O)(OCC)=O
106796	C(CCCCCCCCC(OC)=O)(OC)=O
123728	C(CCC)=O
22819916	N#CCCCCCCCl
109524	C(CCCC)(=O)O
2627272	c1ccccc1CCCN=C=S
609938	c1(c(c([N+](=O)[O-])cc(c1)C)O)[N+](=O)[O-]
3012371	C(#N)SCc1ccccc1

**Table 9:** Analysis of model prediction accuracies for IGC50 for the reduced TPT dataset

**Method name**	**R2**	**RSE**	**Q2**	**MAE**	**RMSE**
NPN	0.84	0.37	0.60	0.44	0.57
PN	0.84	0.37	0.75	0.33	0.46
DMS	0.89	0.30	0.88	0.20	0.32
**3-CPMI**	**0.92**	**0.25**	**0.91**	**0.16**	**0.26**
5-CPMI	0.90	0.28	0.89	0.18	0.29
10-CPMI	0.88	0.32	0.86	0.21	0.33
3-APMI	0.91	0.27	0.90	0.18	0.29
5-APMI	0.90	0.28	0.89	0.19	0.30
Oracle	0.98	0.10	0.98	0.09	0.11

The above examples show the great potential of the model identification methods. We demonstrated that the method based on pre-defined rules (such as maximal similarity for chemicals and minimal error for a model assigned with them) can be compared with the standard machine learning algorithms for the classification problem. Model identification can be considered as an ensemble technique to build high predictive consensus models in predictive toxicology.

### LogP prediction for in-house Syngenta dataset

For the second experiment we considered the estimation of the LogP for an internal Syngenta dataset. The octanol/water Partition coefficient (LogP) is a measure of the lipophilicity of chemical compounds and is an important descriptive parameter in bio-studies [8]. Currently, there are various methods for estimating this coefficient: fragmental methods (CLOGP, KOWWIN), atom contribution methods (TSAR, XLOGP), topological indices (MLOGP), molecular properties (BLOGP).

**Figure 8:**
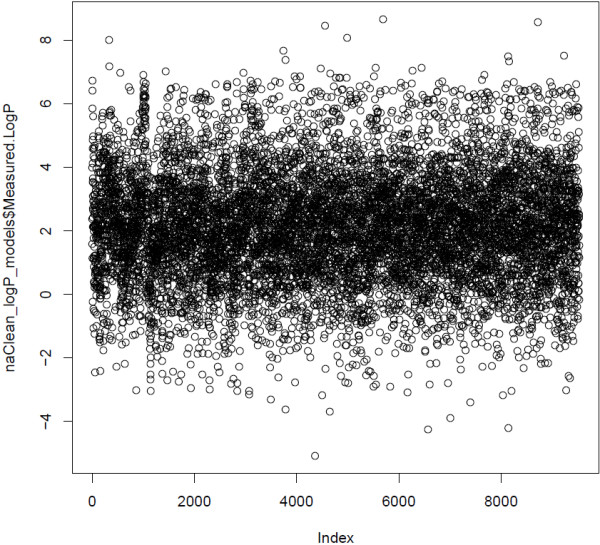
Syngenta measured LogP dataset.

**Figure 9:**
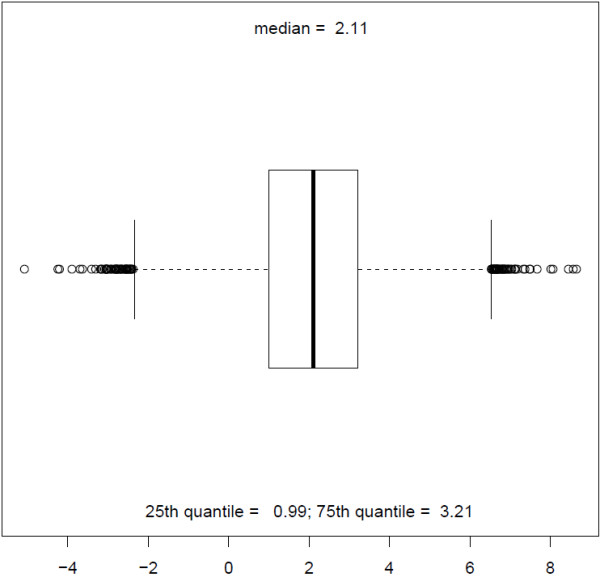
Summary of Syngenta measured LogP dataset.

The initial dataset contains about 9000 chemical compounds and their measured LogP value in Syngenta’s laboratories. The measured value of LogP is in the range [-5.08,8.65] (see Figures 8 and 9). There was no additional data curation than the curation provided by Syngenta researchers. Three models to predict LogP: CLOGP developed in Syngenta, KOWWIN in EPI Suite and MLOGP in Dragon were applied for this dataset. We randomly selected 1000 chemicals (out of 9000) and used the remaining 8000 chemicals as the chemical space of the partitioning model. We used the 3-APMI method as it was the best method in the first experiment. We compared the performance of these four models on 1000 selected chemicals (see Table 10). We repeated the same experiment with 2000 randomly selected chemicals. Additionally, we selected from the initial dataset those chemical compounds for which oracle model has absolute error >0.7. We obtained a set of 2333 chemical compounds.

**Table 10:** Analysis of model prediction accuracies for a LogP estimation

**nr chemicals**	**Mod.Name**	**Q2**	**MAE**	**RMSE**
	CLOGP	0.83	0.38	0.74
1000	MLOGP	0.57	0.84	1.19
	KOWWIN	0.79	0.47	0.83
	3-APMI	0.84	0.38	0.74
	CLOGP	0.76	0.41	0.78
2000	MLOGP	0.44	0.85	1.2
	KOWWIN	0.69	0.50	0.88
	3-APMI	0.78	0.39	0.72
	CLOGP	0.37	1.21	1.54
2333	MLOGP	0.39	1.13	1.52
	KOWWIN	0.41	1.01	1.49
	3-APMI	0.64	0.80	1.16

Table 10 displays the accuracy of model predictions. The 3-APMI is generally at least as good as the best model (CLOGP). In the case of randomly selected chemicals CLOGP was hard to beat, although for 2000 randomly selected chemicals one can clearly see the benefit of using 3-APMI (higher Q2 and lower MAE). The biggest gain is, however, observed for those chemicals whose activity is difficult to predict (the last experiment). This shows that partitioning model (3-APMI) can be a powerful knowledge extraction tool.

All methods proposed in the paper were implemented in R [44]. The log*P* value, fingerprints and Tanimoto similarity were calculated using the RCDK [45] library. A number of tests were run to define the threshold *τ*. It is important to notice that the *n*-Pareto neighbourhood defines the set of at most *n*-Pareto points. Therefore, for the 3-Pareto neighbourhood we found chemicals that have 1, 2, or 3 Pareto neighbours for *τ* = 0.4 for the entire TPT dataset. For the 5-Pareto neighbourhood *τ* = 0.7 and for the 10-Pareto neighbourhood we considered all Pareto neighbours. This shows that a size of the Pareto neighbourhood depends on a size of the available chemical space and may vary for different endpoints. Also, looking at the results for APMI and CPMI one can notice that it is not worth considering all Pareto points, and that the size of the Pareto neighbourhood depends on chemical compound similarities.

## Conclusion

In this paper, we draw attention to advantages of model reusage in predictive toxicology. Since the amount of experimental data and the number of predictive models are growing every day, it is crucial to develop automated methods for mining models in repositories. The most demanding task is to find a model for a new chemical compound from a collection of models for a given endpoint.

In this paper, we proposed two methods (APMI and CPMI) that identify the suitable model for a query chemical compound based on the model performances in its Pareto neighbourhood. These algorithms are based on our simple yet effective method for finding the Pareto set in 2D space. The experimental results demonstrate the advantage of our approach and indicate that automated model identification is a promising research direction with many practical applications. Our approach is mainly focused on regression models and in the future we plan to extend it to classification models, including the analysis of model variables in chemical space partitioning. An additional interesting direction could address the estimation of identified model reliability for a new chemical compound.

## Appendix 1 Proofs

### *Proof*

*(Lemma 1)*. We prove this lemma by contradiction. Let’sj∈{1,…,K} and choose *v* ∈ *Γ*_*j*_. Assume that *v* ∉ *Γ*, which is equivalent to saying that there exists *w* ∈ *V* that is strictly dominated by *v*, i.e. *w* ≺ *v*. This means that *π*_*j*_(*w*) = *π*_*j*_(*v*) and *w* ∈ *V*_*j*_. By the definition of *Γ*_*j*_ we know that *v* is a minimal vector in *V*_*j*_, so *v* ≼ *w*, which contradicts *w* ≺ *v*.

### *Proof*

*(Lemma 2). Let v ∈ V*. First notice that$\pi_{j}(v)\geq f_{j}^{\text{min}}$,j=1,…,K. If$\pi_{j}(v)\notin (f_{j}^{\text{min}},f_{j}^{\text{max}})$ for all *j* then$\pi_{j}(v)\geq f_{j}^{\text{max}}$ for all *j* and *w* ≼ *v* for *w* ∈ I*Γ*. If there exists exactly onej∈{1,…,K} such that$\pi_{j}(v)\in (f_{j}^{\text{min}},f_{j}^{\text{max}})$, then for each index *l* ≠ *j* we have$\pi_{l}(v)\geq f_{l}^{\text{max}}$ and there exists a vector *w* ∈ *Γ*_*j*_ such that *w* ≼ *v*. Therefore, if *v* is incomparable with vectors in I*Γ*, none of the above cases can take place, and the proof is completed.

### *Proof*

*(Lemma 3)*. Notice first that each *Γ*_*j*_, *j* = 1,2, consists of one element, because the Pareto order ≼ induces a linear order on the sets *V*_*j*_. Therefore, I*Γ* consists of at most two elements. Assume that I*Γ* has one element, which we denote by *w*. From the construction of I*Γ* we have:

$$\pi_{1}(w)=f_{1}^{\text{min}},\;\pi_{2}(w)=f_{2}^{\text{min}}.$$

Consequently, *w* is dominated by every vector of *V*, so it is the only minimal vector in *V*.

Assume now that I*Γ* consists of two vectors: *w*_1_ and *w*_2_.

(⇒) After renumbering, *Γ*_1_ = {*w*_1_} and *Γ*_2_ = {*w*_2_}. Hence, we obtain from equations (5)-(7)

$$\begin{array}{ll} f_{1}^{\text{min}}=\pi_{1}(w_{1}), & \;f_{1}^{\text{max}}=\pi_{1}(w_{2}),\\ f_{2}^{\text{min}}=\pi_{2}(w_{2}), & \;f_{2}^{\text{max}}=\pi_{2}(w_{1}). \end{array}$$

Due to (3) the set of vectors *v* ∈ *V* incomparable with I*Γ* satisfies (9).

(⇐) Let *v* ∈ *V* for which inclusion (9) holds, then using renumbering of set *Γ*_*j*_, *j* = 1,2, from the above implication, we obtain:

$$\begin{array}{ll} \pi_{1}(v)>f_{1}^{\text{min}}=\pi_{1}(w_{1}), & \;\pi_{1}(v)<f_{1}^{\text{max}}=\pi_{1}(w_{2}),\\ \pi_{2}(v)<f_{2}^{\text{max}}=\pi_{2}(w_{1}), & \;\pi_{2}(v)>f_{2}^{\text{min}}=\pi_{2}(w_{2}). \end{array}$$

According to the Definition 2 and formula (4) we obtain *v* ∼ *w*_1_ and *v* ∼ *w*_2_. Since I*Γ* = {*w*_1_,*w*_2_}, then *v* is incomparable with the vectors *w*_1_ and *w*_2_.

## Competing interests

The authors declare that they have no competing interests.

## Authors’ contributions

AP proposed the concept of model identification in predictive toxicology. She designed and validated the method that uses Pareto points for model selection from the collection of existing models. She also proposed the algorithm for finding Pareto points in 2D space. DN originated the concept of data and model governance as a framework for reusing and mining models in predictive toxicology. DN, MR have been involved in the review discussions and proofread the draft of this manuscript. All authors have read and approved the final version of the manuscript.
